# Do discrimination, residential school attendance and cultural disruption add to individual-level diabetes risk among Aboriginal people in Canada?

**DOI:** 10.1186/s12889-015-2551-2

**Published:** 2015-12-09

**Authors:** Roland F. Dyck, Chandima Karunanayake, Bonnie Janzen, Josh Lawson, Vivian R. Ramsden, Donna C. Rennie, P. Jenny Gardipy, Laura McCallum, Sylvia Abonyi, James A. Dosman, Jo-Ann Episkenew, Punam Pahwa, on behalf of the First Nations Lung Health Team

**Affiliations:** Canadian Centre for Health and Safety in Agriculture, College of Medicine, University of Saskatchewan, 104 Clinic Place, Saskatoon, Saskatchewan S7N 2Z4 Canada; Department of Medicine, University of Saskatchewan, Saskatoon, Saskatchewan Canada; Department of Community Health and Epidemiology, University of Saskatchewan, Saskatoon, Saskatchewan Canada; Department of Academic Family Medicine, University of Saskatchewan, Saskatoon, Saskatchewan Canada; Willow Cree Health Centre, Duck Lake, Saskatchewan Canada; William Charles Health Centre, Montreal Lake Cree Nation, Saskatchewan Canada; Indigenous Peoples’ Health Research Centre, University of Regina, Regina, Canada

**Keywords:** Colonization, First Nations, Residential school, Racism, Discrimination, Diabetes mellitus, Social determinants of health

## Abstract

**Background:**

Aboriginal peoples in Canada (First Nations, Metis and Inuit) are experiencing an epidemic of diabetes and its complications but little is known about the influence of factors attributed to colonization. The purpose of this study was to investigate the possible role of discrimination, residential school attendance and cultural disruption on diabetes occurrence among First Nations adults.

**Methods:**

This 2012/13 cross sectional survey was conducted in two Saskatchewan First Nations communities comprising 580 households and 1570 adults. In addition to self-reported diabetes, interviewer-administered questionnaires collected information on possible diabetes determinants including widely recognized (e.g. age, sex, lifestyle, social determinants) and colonization-related factors. Clustering effect within households was adjusted using Generalized Estimating Equations.

**Results:**

Responses were obtained from 874 (55.7 %) men and women aged 18 and older living in 406 (70.0 %) households. Diabetes prevalence was 15.8 % among women and 9.7 % among men. In the final models, increasing age and adiposity were significant risk factors for diabetes (e.g. OR 8.72 [95 % CI 4.62; 16.46] for those 50+, and OR 8.97 [95 % CI 3.58; 22.52] for BMI 30+) as was spending most time on-reserve. Residential school attendance and cultural disruption were not predictive of diabetes at an individual level but those experiencing the most discrimination had a lower prevalence of diabetes compared to those who experienced little discrimination (2.4 % versus 13.6 %; OR 0.11 [95 % CI 0.02; 0.50]). Those experiencing the most discrimination were significantly more likely to be married and to have higher incomes.

**Conclusions:**

Known diabetes risk factors were important determinants of diabetes among First Nations people, but residential school attendance and cultural disruption were not predictive of diabetes on an individual level. In contrast, those experiencing the highest levels of discrimination had a low prevalence of diabetes. Although the reasons underlying this latter finding are unclear, it appears to relate to increased engagement with society off-reserve which may lead to an improvement in the social determinants of health. While this may have physical health benefits for First Nations people due to improved socio-economic status and other undefined influences, our findings suggest that this comes at a high emotional price.

## Background

Aboriginal peoples in Canada (First Nations, Inuit and Metis) are experiencing an epidemic of type 2 diabetes (T2DM) [1, 2]. Compared to their non-Aboriginal counterparts, First Nations people not only have higher rates of diabetes [2] but are also more likely to develop diabetes if female [2], as younger adults [2], and during childhood and adolescence [3]. Thus, while average life span is shorter among diabetic First Nations people [4] compared to non-First Nations people, the years lived with diabetes is typically longer [4]. This prolonged exposure to the metabolic consequences of diabetes contributes to a greater risk for chronic complications such as diabetic kidney disease [5, 6], particularly when combined with reduced access to and quality of diabetes care [7, 8]. Understanding the mechanisms underlying these ethnicity-based differences is therefore important in developing effective primary and secondary prevention initiatives and in providing optimal management of diabetes and its complications.

While genetic factors contribute to the risk for T2DM [9], they cannot explain the rapid world-wide emergence of diabetes over the past few decades in diverse populations that include many Indigenous groups [10]. Instead, this pandemic has paralleled recent changes in environmental and possibly epigenetic factors [11] – these have been attributed to unprecedented disruptions in traditional lifestyles that have occurred in virtually all human populations, particularly since the middle of the past century [12]. Most attention has focused on the role of changing diets (e.g. higher consumption of simple carbohydrates and calorie dense foods) and reduced physical activity that have led to a parallel pandemic of overweight/obesity [13]. Indeed, overweight/obesity is an important diabetes risk factor among First Nations people which is compounded by a higher proportion with elevated body mass index (BMI) [14]. There is also mounting evidence that increasing rates of childhood obesity and T2DM are partly driven by an increased incidence of diabetic pregnancies among First Nations women – not only women with gestational diabetes [15] but also their children [16] have a higher risk for T2DM.

In addition to changes in lifestyle, it has become apparent that inequities in the social determinants of health (e.g. poverty, sub-standard housing, low educational attainment, poor food availability, unemployment) [17] are also important predictors of chronic diseases including diabetes [18], and are widespread in Aboriginal communities [19]. These factors are increasingly attributed to the impact of colonization that has severely undermined Indigenous culture and access to resources necessary to improve socio-economic status [20, 21]. In particular, discrimination has been identified as a potent social stressor that may increase vulnerability to physical illness through physiological, psychological and behavioral pathways [22]. The main purpose of this study was to determine if residential school attendance, indicators of cultural disruption and perceptions of discrimination were individual-level predictors of diabetes among adults in two First Nations communities in Canada after adjusting for other recognized diabetes risk factors.

## Methods

This was a cross-sectional survey that examined the prevalence, co-morbidities and both widely recognized and unique predictors of diabetes mellitus among First Nations adults living in two Cree First Nations communities in central Saskatchewan in 2012 and 2013. The study was conducted as part of the First Nations Lung Health Project (FNLHP) that used a community-based participatory research approach. The methodology for the overall study has been described in detail elsewhere [23], but its main features and the specific elements of this research are also summarized here.

The overall project was initially developed through two years of dialogue and ten consultation sessions with community leaders, health workers and community members. Thereafter, a Decision Makers Council consisting of band councilors, elders and youth was formed to oversee the FNLHP. We received letters of agreement from the two communities that included a commitment to follow the Canadian Institutes for Health Research (CIHR) guidelines for conducting research in Aboriginal communities. Regular updates with community representatives are ongoing and all findings are shared with community leaders with agreement to go forward. We have conducted 8 focus groups with communities to share findings and have had 2 national meetings on housing and health led by community leaders. Before conducting the survey, the Biomedical Research Ethics Board of the University of Saskatchewan approved the study (Certificate No. Bio #12-89) and written consent was obtained from all participants.

In developing the baseline questionnaires, feedback was first obtained from community advisors (Elders and health services directors/staff) of both participating communities. Advisors also provided input about the best approaches for contacting participants and for collecting questionnaire and clinical assessment data. A pilot study was conducted to optimize the content and administration of the baseline questionnaires. Based on the pilot project responses, several questions were modified in the final survey. In both First Nations communities, the baseline assessment consisted of two stages carried out by trained community research assistants. The first stage involved door-to-door canvassing to advertise the survey by distributing brochures and explaining the need and purpose of the study. For the second stage, every adult (18 years and older) was invited to visit the community health care center to complete interviewer-administered questionnaires and to participate in clinical assessments [23] (height, weight, waist circumference, blood pressure, pulmonary function and allergy skin prick tests). Finally, an identification number was assigned to each household using the community map, and a key informant from each household was asked to provide household level information. The sampling frame and sample size calculations can be found in our Methods paper [23].

### Primary health outcome

The questionnaires were primarily designed to obtain information on the individual and contextual determinants of respiratory health, but also included questions pertaining to general health and other health conditions. Accordingly, the outcome of interest for this study was self-reported physician-diagnosed diabetes mellitus. We did not attempt to distinguish between type 1 and type 2 diabetes.

### Contextual factors

The contextual factors of interest in this sub-study study were: housing environment, socioeconomic status, household income, and factors that can be attributed to colonization [20, 21]. This was approached by asking questions about knowledge of Indigenous language (Cree), participation in traditional cultural activities, scoring of community strengths (scale 1–12), residential school experience [24] (either personally or by family members), and perceptions of discrimination. The latter variable was created by summing the affirmative responses to 11 situations modified from the 9 item scale published by Krieger [25–27] (Have you ever experienced discrimination or racism, been prevented from doing something, or been hassled or made to feel inferior in any of the following situations because of your race, cultural group or color: *at school; getting hired or getting a job; at work; getting housing; getting medical care; getting medicine at pharmacies; getting service in a store or restaurant; getting credit, bank loans or mortgage; on the street or public setting; from the police or the courts; getting a check cashed*). Affirmative responses were divided into three categories of perceived discrimination: low (0–3), moderate (4–7), high (8–11). These differed from Krieger’s cut-offs (0, 1 or 2, 3–9 respectively) because of the additional 2 items (*getting medicine at pharmacies; getting a check cashed)* and to more clearly identify those experiencing the most discrimination.

Following an extensive review of existing measures of discrimination, we chose the Krieger scale because of its established psychometric properties and its brevity compared to similar scales. Although extensive validation procedures were not conducted (as we did not have the data to do so), pilot testing of the instrument indicated that the scale items resonated with community members. Furthermore, to further enhance the content validity of the scale, two items were added based on community feedback (see above). We were also able to determine that the scale had high internal consistency in our sample (Cronbach alpha = .87), an important component when assessing the validity of a scale. Nonetheless, additional research is needed to further examine its’ validity and reliability in relation to the experiences of Indigenous people in a Canadian context.

### Individual factors

The individual factors considered were: lifestyle or behavior-related factors (smoking, alcohol use, body mass index (BMI); waist circumference, and physical activity); and individual educational attainment.

### Covariates

Information was obtained on important covariates such as age, sex and marital status. There were also questions pertaining to perceptions of general health as well as selected co-morbid conditions including depression, hypertension, macro-vascular disease (heart disease, stroke, leg ulcers, amputation), micro-vascular disease (eye sight problems and kidney disease), tuberculosis, cancer and several indicators of chronic lung disease.

### Statistical analysis

Statistical analyses were conducted using SPSS version 22 (SPSS Inc. Armonk, NY: IBM Corp.). For baseline survey data, frequencies were computed for all variables. Both crude and age specific diabetes prevalence were presented as observed/total and percentage. Chi-square tests were used to determine the univariate association of diabetes prevalence with the independent variables of interest. Logistic regression models were used to predict the relationship between a binary diagnosis of diabetes (yes or no) and a set of explanatory variables. A multilevel logistic regression modeling approach based on a generalized estimating equations, with individuals (1st level) nested within households (2nd level), was utilized to evaluate the effects of both contextual and individual factors after adjustment for covariates of interest. This accounts for the within subject dependencies that occur in the analysis due to multiple people from the same household. A series of multi-level models were fitted to determine whether potential risk factors, confounders, and interactive effects (e.g. individual and contextual risk factors) contributed significantly to the prevalence of diabetes. Based on bi-variable analysis, variables with *p* < 0.20 were candidates for the multivariate model. All variables that were statistically significant (*p* < 0.05) as well as important contextual factors (discrimination), were retained in the final multivariable model. Interactions between potential effect modifiers were examined and were retained in the final model if the p-value was <0.05. The predicted probability graphs were prepared for significant interactions. A parsimonious model was selected based on QIC (Quasi likelihood under the Independence model Criteria) goodness-of-fit statistic [28, 29]. The strengths of associations were presented by odds ratios (OR) and their 95 % confidence intervals (CI).

## Results

Of the 1570 eligible adults (18 years of age and older) and 580 eligible households in the two First Nations communities, 874 (55.7 %) individuals living in 406 (70 %) households participated in the survey (Table 1). Of those, 443 (51 %) were female and 431 (49 %) were male; their mean age was 35 years (range 18–85 plus 1 person aged 17). There were 112 people with diabetes (42 males and 70 females). The overall crude diabetes prevalence was 12.8 % with a higher proportion of affected females than males (15.8 % versus 9.7 %). The age-specific prevalence ranged from 3.6 % in those under 30 years of age to 32.6 % among those 50 and older.

**Table 1:** Study population characteristics – adults 18 and older^a^

Parameters	Number	Percent
Eligible Households	580	-
Participating Households	406	70
Eligible Adults	1570	-
Participating Adults (total)	874	55.7
Men	431	49.3
Women	443	50.7
Total self-reported diabetes	112	12.8
Men	42	9.7
Women	70	15.8
Diabetes by Age Group (Total)		
50 and older	56 (172)	32.6
40–49	23 (135)	17.0
30–39	19 (175)	10.9
Under 30	14 (392)	3.6

^a^The age/sex distribution of the study population closely approximated the 2011Canadian census for these two communities

Table 2 shows the univariate relationships between key variables and diabetes. Those with diabetes were more likely to be female, older, divorced/separated/widowed, and to be retired/homemaker/disabled. Single people and those who were either employed or students were less likely to have diabetes. In this study, lower educational attainment and reduced household income were not predictors of diabetes although the prevalence was significantly higher among people living in houses requiring major repairs. First Nations people who were overweight, obese or had an increased abdominal girth were respectively 5.9, 14.5 and 8.5 times more likely to have diabetes than those with normal adiposity. Regular alcohol intake and being a current smoker both significantly reduced diabetes risk in univariate analysis but not after adjusting for other independent variables. There was a trend for exercise to reduce diabetes risk in a dose-dependent manner but this relationship was not statistically significant.

**Table 2:** Univariate associations between diabetes and individual characteristics

Parameter	Diabetes Prevalence (%)	Odds Ratio (95 % CI)	Parameter	Diabetes Prevalence (%)	Odds Ratio (95 % CI)
Sex			Life Style		
Female	15.8	1.73 (1.16, 2.58)	Body Mass Index		
Male	9.7	1.00 (ref)	Obese	23.8	14.5 (6.09, 34.61)
			Overweight	11.2	5.86 (2.40, 14.35)
Age			Normal	2.1	1.00 (ref)
50+ years	32.6	13.5 (7.27, 24.98)			
40-49 years	17.0	5.63 (2.79, 11.35)	Abdominal Girth^a^		
30-39 years	10.9	3.39 (1.69, 6.78)	High risk	19.2	8.50 (4.06, 17.79)
17-29 years	3.6	1.00 (ref)	Not available	16.1	5.73 (2.35, 13.97)
			Low risk	2.7	1.00 (ref)
Marital Status					
Single	8.4	0.47 (0.31, 0.74)	Smoking Status		
Divorced/separated/widowed	29.8	2.19 (1.21, 3.94)	Current smoker	10.9	0.53 (0.30, 0.95)
Married/common law	16.3	1.00 (ref)	Ex-smoker	20.2	1.10 (0.56, 2.15)
			Never Smoker	18.6	1.00 (ref)
Education Attained					
Less than high school	12.8	0.99 (0.66, 1.49)	Alcohol Intake^b^		
High school or higher	12.9	1.00 (ref)	Regular	8.8	0.58 (0.35, 0.98)
			Never or not regular	14.2	1.00 (ref)
Household income					
$0- $9,999	12.2	0.92 (0.55, 1.56)	Exercise		
$10,000- $19,999	12.6	0.96 (0.49, 1.85)	Yes	11.9	0.78 (0.51, 1.19)
Refused/Not stated	13.2	1.01 (0.60, 1.69)	No	14.9	1.00 (ref)
$20,000+	13.0	1.00 (ref)			
			Duration of Exercise		
Employment Status			0 - <15 minutes	15.1	1.16 (0.66, 2.05)
Employed (full/part/student)	9.6	1.00 (ref)	15-30 minutes	10.8	0.79 (0.42, 1.50)
Retired/homemaker/disabled	20.2	2.38 (1.41, 4.04)	31-60 minutes	9.8	0.71 (0.36, 1.40)
Unemployed	11.6	1.23 (0.74, 2.04)	>60 minutes	13.3	1.00 (ref)
House Environment			Sleep Hours		
Crowding			More than 7 hours	11.4	0.77 (0.43, 1.40)
<1 person/bedroom	19.5	1.87 (1.04, 3.36)	6-7 hours	14.0	0.99 (0.56, 1.76)
1 person/bedroom	15.5	1.41 (0.85, 2.34)	5 hours or less	14.3	1.00 (ref)
>1 person/bedroom	11.5	1.00 (ref)			
House needs repair					
Yes, major repairs	15.7	1.66 (1.00, 2.74)			
Yes, minor repairs	12.5	1.28 (0.72, 2.25)			
No, only regular maintenance	10.4	1.00 (ref)			

^a^High risk if abdominal girth ≥102 cm for men; ^a^High risk if abdominal girth ≥88 cm for women

^b^Regular was defined as alcohol intake of once a week or more

Table 3 shows the relationship between diabetes and factors that can be attributed to colonization. Diabetes prevalence was slightly higher among participants who had attended residential schools than those who had not (13.5 % versus 12.4 %) but the difference was not statistically significant (OR 1.10; 95 % CI 0.74, 1.66). Similarly, having a parent or grandparent who had attended residential school did not significantly predict diabetes among study participants, although the overall number of community members who did not have an inter-generational family exposure to residential schools was <10 %. Additional analyses (not shown) that examined the relationship between diabetes and residential school attendance by sex, age group, micro/macro-vascular complications and community, also revealed no statistically significant relationships. The degree to which people participated in cultural events or perceived community strength were also neither predictive nor protective in their relationship with diabetes. In contrast, those who spoke Cree or lived exclusively in their home communities were significantly more likely to have diabetes than those who spent some time off-reserve. However, ability to speak Cree was not a significant predictor of diabetes after adjusting for other independent variables.

**Table 3:** Univariate associations between diabetes and factors related to colonization

Parameter	Diabetes Prevalence (%)	Odds Ratio (95 % CI)	Parameter	Diabetes Prevalence (%)	Odds Ratio (95 % CI)
Residential School Attendance					
Study participant?			Take part in cultural events?		
Yes	13.5	1.10 (0.74, 1.66)	Always/almost always	12.3	1.08 (0.45, 2.60)
No	12.4	1.00 (ref)	Sometimes	14.3	1.29 (0.60, 2.78)
			Rarely	9.8	0.84 (0.34, 2.12)
Parents/grandparents?			Never	11.4	1.00 (ref)
Yes	12.2	0.65 (0.36, 1.19)			
Do not know	13.3	0.72 (0.28, 1.85)	Community Strength^b^		
No	17.4	1.00 (ref)	Low	14.2	1.28 (0.85, 1.95)
			High	11.4	1.00 (ref)
Experience discrimination^a^					
High (8–11)	2.4	0.15 (0.03, 0.65)	Past Year Spent on Reserve		
Moderate (4–7)	15.1	1.15 (0.70, 1.87)	All 12 months	15.5	2.46 (1.48, 4.08)
Little (0–3)	13.6	1.00 (ref)	Less than 12 months	7.0	1.00 (ref)
Language					
Can speak Cree	21.2	3.65 (2.37, 5.64)			
Cannot speak Cree	6.9	1.00 (ref)			

^a^This variable was created by summing up the affirmative responses to 11 situations (see methods)

^b^This variable was created by summing up the affirmative responses to 12 strengths in the community. If the study participant perceived less than or equal to 4 community strengths it was coded as low, and if greater than 4 community strengths it was coded as high

Table 3 also shows the association between perceptions of discrimination and diabetes prevalence. Those experiencing the highest level of discrimination had the lowest prevalence of diabetes when compared to those experiencing little discrimination (2.7 % versus 13.6 %; OR 0.15; 95 % CI 0.03, 0.55). Those exposed to a moderate level of discrimination had a slightly higher prevalence of diabetes than the reference group but the difference was not statistically significant.

The univariate associations between diabetes and several important co-morbidities are shown in Table 4. Overall, people with diabetes were 3.6 times more likely to perceive their general health as fair and over 4 times as likely to perceive it as poor compared to those without diabetes. These perceptions paralleled the presence of micro-vascular and macro-vascular disease (ORs 3.63 and 4.25 respectively). People with diabetes were also more likely to report a significant history of cancer, tuberculosis and asthma. Although chronic respiratory diseases and obstructive sleep apnea were more prevalent in people with diabetes, the findings did not achieve statistical significance. Nonetheless, symptoms such as shortness of breath, snoring and abnormal sleepiness were significantly associated with diabetes, as were several tests indicating poorer lung function. Finally, there was a trend for people with diabetes to have higher blood pressures, mental health problems and depression but once again the findings were not statistically significant.

**Table 4:** Univariate associations between diabetes and co-morbidities

Parameter	Diabetes Prevalence (%)	Odds Ratio (95 % CI)	Parameter	Diabetes Prevalence (%)	Odds Ratio (95 % CI)
Shortness of Breath			Tuberculosis		
Yes	16.7	2.17 (1.40, 3.36)	Yes	22.0	2.08 (1.07, 4.03)
No	8.5	1.00 (ref)	No	12.0	1.00 (ref)
Asthma			Sleep Apnea		
Yes	19.5	1.87 (1.17, 2.98)	Yes	19.4	1.73 (0.89, 3.36)
No	11.4	1.00 (ref)	No	12.2	1.00 (ref)
General Physical Health			Chronic Respiratory Diseases^c^		
Poor	22.7	4.11 (1.19, 14.26)	Yes	21.2	1.95 (0.96, 3.99)
Fair	20.5	3.61 (1.28, 10.20)	No	12.1	1.00 (ref)
Good	11.3	1.78 (0.61, 5.17)			
Very good	7.3	1.10 (0.34, 3.54)	Snore		
Excellent	6.7	1.00 (ref)	Yes	15.2	1.64 (1.07, 2.53)
			Do not know	12.3	1.28 (0.71, 2.31)
Mental Health			No	9.9	1.00 (ref)
Fair to poor	18.5	1.68 (0.98, 2.89)			
Excellent to good	11.9	1.00 (ref)	Snore Volume		
			Loud to very loud	21.0	2.43 (1.41, 4.19)
Depression			Softer than talking	10.6	1.08 (0.59, 1.98)
Yes	16.8	1.54 (0.95, 2.50)	Do not know	12.9	1.35 (0.81, 2.26)
No	11.5	1.00 (ref)	Never	9.9	1.00 (ref)
Macro Vascular Complications^a^			Epworth Sleepiness Scale		
Yes	32.1	4.25 (2.67, 6.77)	Abnormal	17.6	1.73 (0.99, 3.03)
No	10.1	1.00 (ref)	Normal	11.0	1.00 (ref)
Micro Vascular Complications^b^			Systolic Blood Pressure		
Yes	29.0	3.63 (2.33, 5.66)	High	15.4	1.44 (0.93, 2.22)
No	10.1	1.00 (ref)	Not Available	12.7	1.16 (0.59, 2.29)
			Low	11.3	1.00 (ref)
Cancer					
Yes	31.0	3.32 (1.49, 7.40)			
No	11.9	1.00 (ref)			
Lung function measurements	Mean ± SD	Odds Ratio (95 % CI)			
FVC					
Diabetes-Yes	3.8 ± 1.0	0.45 (0.34, 0.60)			
Diabetes-No	4.6 ± 1.1	1.00 (ref)			
FEV_1_					
Diabetes-Yes	3.0 ± 0.8	0.38 (0.28, 0.52)			
Diabetes-No	3.8 ± 0.9	1.00 (ref)			
FEV_1_/FVC ratio %					
Diabetes-Yes	80.5 ± 7.4	0.97 (0.94, 0.99)			
Diabetes-No	82.7 ± 7.2	1.00 (ref)			

^a^This included heart disease, stroke and leg ulcers or amputations

^b^This included eye sight problems and kidney problems

^c^This included chronic bronchitis, emphysema and chronic obstructive pulmonary disease (COPD)

Tables 5 show results from two models of diabetes risk based on multivariate logistic regression analysis. The first was carried out with and without BMI included in the model; and the second with and without abdominal girth. In both models the results were similar. Increasing age and increasing adiposity (either BMI or high risk abdominal girth) were highly predictive of diabetes when measures of adiposity were included in the model, while female sex was not. In contrast, when BMI and abdominal girth were removed from the model, women were over 50 % more likely to have diabetes than men (OR 1.54; 95 % CI 1.00, 2.38).

**Table 5:** ORs of diabetes risk based on multivariate logistic regression analysis

Model with BMI			Model without BMI		
Parameter	Odds Ratio (95 % CI)	*P* value	Parameter	Odds Ratio (95 % CI)	*P* value
Sex			Sex		
Female	1.21 (0.78, 1.88)	0.397	Female	1.54 (1.00, 2.38)	0.050
Male	1.00 (ref)		Male	1.00 (ref)	
Age			Age		
50+ years	8.72 (4.62, 16.46)	<0.0001	50+ years	12.82 (6.94, 23.67)	<0.0001
40–49 years	3.62 (1.78, 7.35)	<0.0001	40-49 years	5.15 (2.58, 10.27)	<0.0001
30–39 years	2.44 (1.19, 4.99)	0.015	30-39 years	3.28 (1.63, 6.58)	0.001
17–29 years	1.00 (ref)		17-29 years	1.00 (ref)	
Body Mass Index					
Obese	8.97 (3.58, 22.52)	<0.0001			
Overweight	3.86 (1.51, 9.87)	0.005			
Normal	1.00 (ref)				
Past year duration in reserve			Past year duration in reserve		
All 12 months	1.69 (0.96, 2.97)	0.068	All 12 months	1.83 (1.05, 3.18)	0.032
Less than 12 months	1.00 (ref)		Less than 12 months	1.00 (ref)	
Ever experienced discrimination			Ever experienced discrimination		
High (8–11)	0.11 (0.02, 0.54)	0.007	High (8–11)	0.12 (0.03, 0.54)	0.006
Moderate (4–7)	1.23 (0.70, 2.16)	0.464	Moderate (4–7)	1.24 (0.73, 2.13)	0.428
Little (0–3)	1.00 (ref)		Little (0–3)	1.00 (ref)	
Model with Abdominal Girth			Model without Abdominal Girth		
Parameter	Odds Ratio (95 % CI)	*P* value	Parameter	Odds Ratio (95 % CI)	*P* value
Sex			Sex		
Female	1.01 (0.64, 1.58)	0.978	Female	1.54 (1.00, 2.38)	0.050
Male	1.00 (ref)		Male	1.00 (ref)	
Age			Age		
50+ years	9.87 (5.29, 18.41)	<0.0001	50+ years	12.82 (6.94, 23.67)	<0.0001
40–49 years	4.12 (2.01, 8.46)	<0.0001	40-49 years	5.15 (2.58, 10.27)	<0.0001
30–39 years	2.64 (1.28, 5.46)	0.009	30-39 years	3.28 (1.63, 6.58)	0.001
17–29 years	1.00 (ref)		17-29 years	1.00 (ref)	
Abdominal Girth					
High risk	5.87 (2.67, 12.92)	<0.0001			
Not available	4.40 (1.75, 11.07)	0.002			
Low risk	1.00 (ref)				
Past year duration in reserve			Past year duration in reserve		
All 12 months	1.84 (1.05, 3.20)	0.032	All 12 months	1.83 (1.05, 3.18)	0.032
Less than 12 months	1.00 (ref)		Less than 12 months	1.00 (ref)	
Ever experienced discrimination			Ever experienced discrimination		
High (8–11)	0.11 (0.02, 0.49)	0.004	High (8–11)	0.12 (0.03, 0.54)	0.006
Moderate (4–7)	1.11 (0.63, 1.93)	0.717	Moderate (4–7)	1.24 (0.73, 2.13)	0.428
Little (0–3)	1.00 (ref)		Little (0–3)	1.00 (ref)	

As in univariate analysis (above), place of habitation continued to be a significant predictor of diabetes in 3 of the 4 multivariate models. Thus, those living on-reserve for all 12 months of the year were more likely to have diabetes than those who spent at least part of the time off-reserve. Finally, those experiencing the highest levels of discrimination experienced a significantly lower risk of diabetes (OR 0.11; 95 % CI 0.02, 0.54) after adjusting for other key variables such as sex, BMI (or waist circumference) and age group. Removal of adiposity measures from the analysis did not affect this finding. However, we additionally looked for possible trends in other variables that might explain the inverse relationship between diabetes and perceptions of discrimination (data not shown). Compared to those with the lowest levels of perceived discrimination, a larger proportion of people with the highest levels of perceived discrimination were employed or students (41 % versus 34.1 %; *p* = 0.791), married or common-law (53 % versus 37.3 %; *p* = 0.007), had household incomes > $20,000 (38.6 % versus 23.1 %; *p* = 0.022), and had attended residential schools (68.7 % versus 35.7 %; *p* < 0.0001).

## Discussion

The prevalence of diabetes among adults in two Saskatchewan First Nations communities was higher than that reported for the province’s non-First Nations population [2], as well as for a predominantly Caucasian sub-group living in rural Saskatchewan during a similar time period [30]. As expected, we found that increasing age and body weight were strong predictors of diabetes in these First Nations communities. We again confirmed the marked predilection of diabetes for First Nations females [2] and have now shown that this is at least partly explained by increased adiposity. With respect to the primary objective of the study, we were unable to show at the individual level that residential school attendance by either study participants or their parents/grandparents significantly increased diabetes risk. The same was true for inability to speak Cree and/or low participation in cultural events. The most unique finding of this study was that those experiencing the highest levels of discrimination at an individual level had the lowest prevalence of diabetes.

As far as we are aware, this is the first study that has examined the relationship between individual-level diabetes risk and factors related to colonization within First Nations communities, while adjusting for a comprehensive set of other important and recognized predictors of diabetes. In addition to female gender, increasing age, higher BMI and elevated waist circumference, known diabetes risk factors include social determinants of health [17, 18] such as poorer income, employment status, education and quality of housing. In our study, better housing and being employed or engaged as a student were associated with lower diabetes prevalence on univariate analysis, but did not remain significant in the final model. Higher household income adequacy and education were not protective for diabetes before or after adjusting for other variables. However, it is possible that we did not sufficiently discriminate between the highest and lowest levels of education and income, and/or that participant numbers in the study did not afford us the statistical power to show a difference. With respect to life style, univariate analysis showed a trend towards lower diabetes prevalence among those who engaged in physical activity, and there were lower rates of diabetes among those who currently smoked or consumed alcohol on a regular basis. In other studies, alcohol intake has been shown to protect against diabetes [31] while smoking is an important diabetes risk factor [32]. Since neither activity remained in the final model in this study, it is possible that our univariate findings were related to higher alcohol intake and smoking rates among younger people who have a much lower prevalence of diabetes.

Results of qualitative research have consistently identified various dimensions of cultural continuity/disruption as important to the health of Indigenous Canadians [33, 34]. However, the results of quantitative research on this topic are mixed. On the one hand, several quantitative studies have demonstrated associations between greater cultural continuity and better mental health outcomes [35, 36]. For example, Chandler and Lalonde have shown that cultural continuity in First Nations communities is protective against youth suicide [37, 38]. In addition, preliminary research conducted as part of the larger study from which our data is drawn, also shows a significant relationship between discriminatory experiences and compromised mental health among First Nations adults [39]. However, a number of other quantitative studies in Canada have reported either no or mixed/contradictory associations between indicators of cultural continuity and health, depending upon the health outcome and particular aspect of culture under study [40–42]. With respect to metabolic disorders, there is only one study that suggests that cultural continuity may be protective against diabetes for First Nations people [43]. After adjusting for differences in income, employment rates and education in 31 First Nations communities in Alberta, Oster and colleagues recently reported on an ecologic study that showed lower diabetes prevalence in those communities that had a greater retention of Indigenous language.

In this study, lack of participation in cultural events and low scoring of community strengths was neither predictive nor protective for diabetes on an individual level. People who spoke Cree were over 3 times more likely to have diabetes than others on univariate analysis but this did not remain significant in the final model. It is likely that the univariate finding was due to a larger proportion of older people with a knowledge of Cree, since increasing age is also a strong predictor of diabetes. It should be emphasized that an important difference between this study and that of Oster et al. mentioned above [43], is that we examined the individual-level risk of diabetes by his/her knowledge of Cree. In contrast, Oster et al. examined the relationship between diabetes prevalence and Indigenous language knowledge at the community level. At face value, these findings may appear contradictory. However, had they looked at within-community data (and regardless of a particular community’s prevalence of diabetes), it is quite possible that Oster et al. would have also found a higher proportion of older individuals with diabetes, and it is also quite possible that older diabetic individuals were more likely to speak an Indigenous language than their younger counterparts.

Although study participants who had attended residential school had a slightly higher prevalence of diabetes than those who had not, the finding was not statistically significant. Furthermore, while those whose parents or grandparents had attended residential school trended towards a lower prevalence of diabetes than participants without that family history, the difference was also not significant. Because of the stressful and often traumatic experiences that many residential school survivors report [24], as well as the intra- and inter-generational impact of those experiences [44], we had anticipated that residential school attendees and their offspring might have a higher risk for diabetes. It is known that stress can be diabetogenic through many pathways [22] including endocrine mechanisms [45] and by causing chromosomal damage [46]. However, it is possible that the detrimental impact of residential schools on the health of individuals within First Nations communities is so pervasive that it is difficult to find a truly unexposed comparison group. Furthermore, there are many additional causes of stress within First Nations communities and it may not be possible to readily differentiate between the relative importance of residential school exposure and other stress-producing experiences. Larger study populations and/or comparison with non-First Nations groups truly unexposed to the impact of residential schools should be considered in future research examining this question.

We had also speculated that those experiencing the most discrimination would have a higher prevalence of diabetes, once again because of increased stress. Instead, we found the opposite. The extent to which people perceived discrimination during day-to day experiences was inversely related to diabetes prevalence, and this highly significant finding was present even after adjusting for other important independent variables. What could explain this relationship? It was not because of characteristics most clearly associated with lower diabetes prevalence such as younger age, being male and having normal adiposity. It also seems unlikely that avoidance of the health care system and delay of diabetes diagnosis could explain such a significant finding. Rather, the main thread that appears to connect those within this group is that they were experiencing negative interactions in what were predominantly non-Aboriginal settings. Indeed, our multivariate model results showed that spending time away from the reserve was protective for diabetes. Finally, and consistent with recent research involving Indigenous Australian adults [47, 48], we also observed that people experiencing the most discrimination (and lowest diabetes prevalence) tended to be employed or students, and were significantly more likely to be married and to have higher household incomes. It is therefore possible that experiencing discrimination is a proxy measure for being engaged in and with society off-reserve. That engagement may lead to improvements in the social determinants of health - including higher socio-economic status - and engender physical health benefits, at least with respect to diabetes risk. As discussed above, however, this comes at a high emotional price via increased experiences of interpersonal discrimination [39]. This is perhaps the most important finding of our study and requires further reflection as well as corroboration to fully understand its implications. As with the impact of residential schools, comparison of First Nations with non-First Nations populations, while adjusting for social determinants of health, could clarify the links between discrimination and physical health outcomes like diabetes.

First Nations people with diabetes were more likely than others to experience a number of co-morbidities and to evaluate their general health as fair or poor. Because the primary focus of the main study was on lung health [23], much of the questionnaire pertained to pulmonary conditions, and many significant associations of these with diabetes were present on univariate analysis. While none were significant in initial multivariate models that included co-morbidities (perhaps largely due to age adjustment), it is likely that obstructive sleep apnea is common among First Nations people with diabetes because obesity is an important risk factor for both [49]. Not surprisingly, both macro and micro vascular complications were very common among those with diabetes and these relationships remained highly significant in initial multivariate models that included co-morbidities. Interestingly, women with diabetes were less likely than men to have micro-vascular complications like kidney and eye disease. This is consistent with other research that shows a higher cumulative incidence of end stage renal disease among First Nations men [4].

Strengths of this study included the community-based participatory research model used, the large proportion of eligible adults and households that participated, and our ability to investigate the potential role of both known and unrecognized variables in understanding the disproportionate risk for diabetes experienced by First Nations communities. Accordingly, this is the first study that we are aware of to examine the potential role of factors related to colonization in diabetes risk while at the same time adjusting for a comprehensive set of other known diabetes predictors. Limitations include the cross sectional nature of the study and the associated inability to be confident about cause-effect relationships. To limit the length of the questionnaire, we omitted questions on important predictors of diabetes such as family history. Although the sample size was sufficient for the main study, the relatively small number of people with diabetes in this sub-study may have prevented us from demonstrating some relationships that truly exist, most importantly the impact of residential school attendance on diabetes risk. The discrimination scale that was used reflects racism on a personal level and we were not able to study the impact of other forms of racism [50] on diabetes risk. For example, it is quite possible that some elements of systemic racism experienced by First Nations people might be more clearly associated with diabetes risk. This reinforces the need for more research focusing on quantitative measurements of cultural disruption, residential school impact and racism among Indigenous peoples. Finally, while our sample had an equal gender distribution, it contained a relatively larger proportion of older people compared to the overall Saskatchewan First Nations population [51]. Therefore, we may not be able to generalize our findings to other First Nations communities.

## Conclusions

Increased diabetes prevalence in two Saskatchewan First Nations communities was significantly related to known diabetes risk factors including female sex, increasing age and BMI but we were unable to demonstrate in this study that diabetes was associated with residential school exposure, perceptions of community strength or inability to speak Cree. An unanticipated finding was that those experiencing the most discrimination had the lowest prevalence of diabetes. While the reasons underlying this latter finding remain unclear, engagement with society off-reserve may have certain physical health benefits for First Nations people that relate to improved socio-economic status and other undefined influences. However, our findings suggest that better physical health comes at a high emotional price via increased experiences of interpersonal discrimination [39]. While this important observation may apply to other Indigenous peoples in Canada and elsewhere, it requires corroboration in diverse jurisdictions. Additional dialogue with the communities as well as further reflection and in-depth analysis will help to fully understand its implications.

### Availability of data and materials

The data used in the analysis is kept in a secure location within the Canadian Center for Health and Safety in Agriculture, University of Saskatchewan. As part of the contractual agreement with the two First Nations communities involved in the study, the data is not publicly available.
